# Modified Unna Boot and Pinch Grafting for Chronic Non-healing Venous Leg Ulcer

**DOI:** 10.4103/0974-2077.41155

**Published:** 2008-01

**Authors:** D N Balaraju, Chakravarthi R Srinivas, Sanjay V Mukhi

**Affiliations:** *Department of Dermatology, PSG Institute of Medical Sciences and Research, Coimbatore, Tamil nadu, India*

**Keywords:** Glycerine, Unna’s boot, venous ulcer, zinc oxide

## Abstract

Venous ulcers cause considerable morbidity. A 45-year-old man reported with non healing ulcer since 15 years. Patch tests revealed multiple sensitivity. Infection was first controlled with antibiotics and antiseptics. Dressing with modified Unna’s boot made with zinc oxide 40% and glycerine paste 60% resulted in formation of healthy granulation tissue. Pinch grafting was subsequently done to promote epithelization. We recommend the use of Unna’s paste instead of more expensive synthetic and occlusive and semi occlusive dressing to promote granulation tissue and pinch grafting after bed is ready to hasten epithelization.

## INTRODUCTION

Venous ulcers are the most common form of leg ulcers. These ulcers have perplexed the surgeons and the dermatologists alike. Although the various treatment modalities are available, none of them assures complete cure, unless the primary cause is tackled. We report a case of chronic venous leg ulcer treated with Unna boot and pinch grafting.

## CASE REPORT

**Figure 1:**
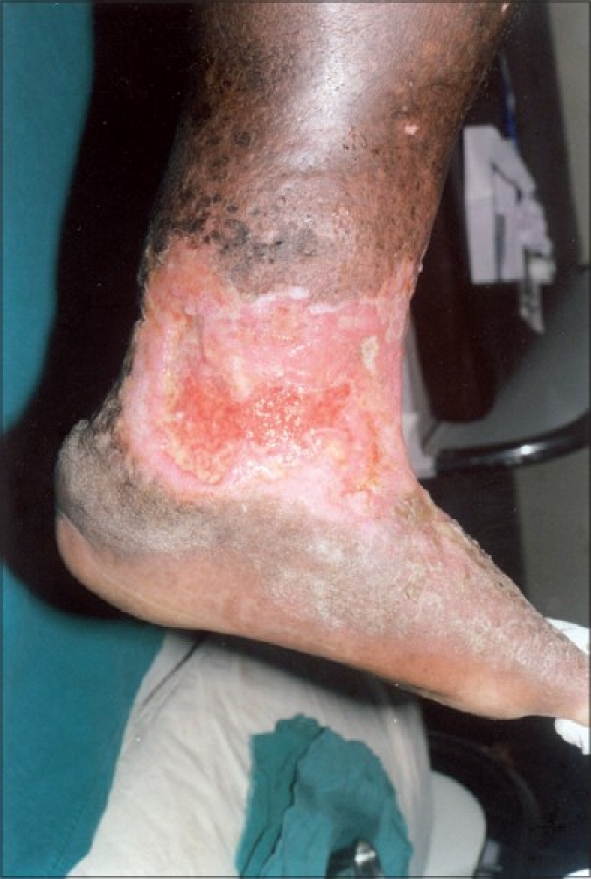
Irregular, non-healing ulcer on the lower leg and ankle with unhealthy granulation tissue

A 57-year-old male presented with a non-healing ulcer over the left leg of 15 years duration [Figure 1]. The ulcer was 15 × 8 cm^2^ in size with irregular borders and indurated base. Lipodermatosclerosis was seen in the surrounding skin. Doppler study revealed venous insufficiency, incompetent perforators in the mid-thigh and sapheno-femoral reflux.

Patch test with Indian standard series and interpreted as recommended by International Contact Dermatitis Research Group (ICDRG), was positive for potassium dichromate, neomycin sulphate, formaldehyde, parabens, colophony, propylene glycol, mercapto-mix, epoxy resin, fragrance mix, nitrofurozone, balsam of Peru, thiuram mix, chinoform and black rubber mix.

Investigations such as complete blood picture and urine routine were within normal limits.

**Figure 2:**
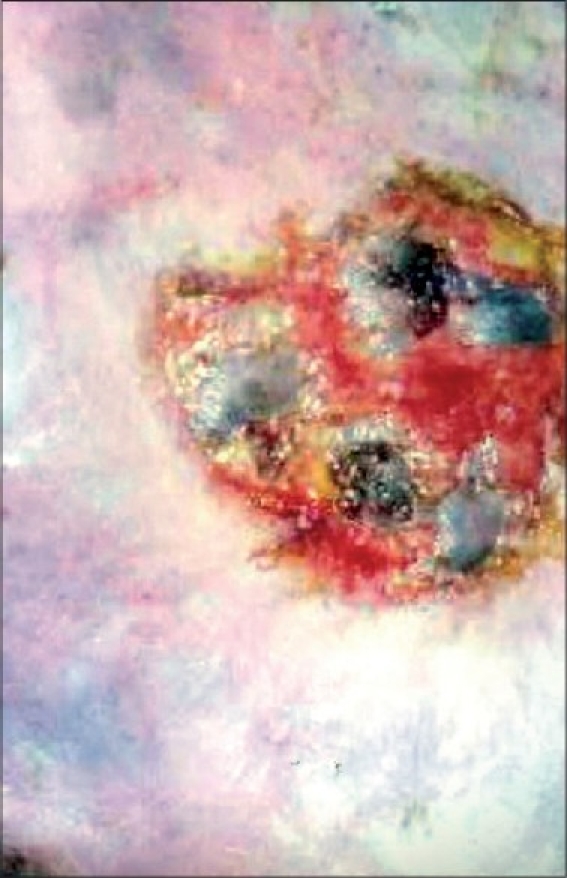
Pinch grafting done after 1 week of modified Unna Boot dressing

**Figure 3:**
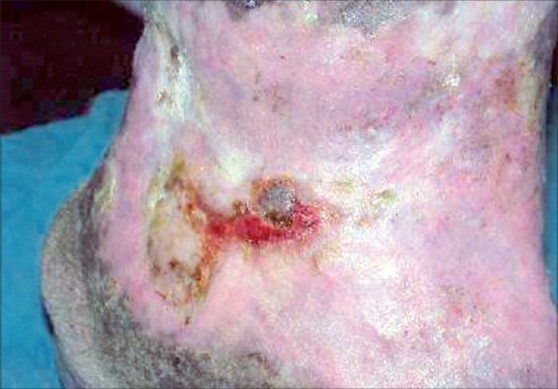
Ulcer is almost completely healed after pinch grafting

Patient was treated with antibiotics based on culture and sensitivity report and daily dressing of ulcer with saline and povidone iodine, till the infection was controlled. The modified Unna boot, made with moist 40% zinc oxide and 60% glycerin paste was spread over a gauze pad and was applied directly over the wound. Above this a roller gauze pad was placed and pressure reinforced with elasto-crepe bandage. This was kept in place for 7 days, removed and re-applied. One week later base was seen covered with healthy granulation tissue and pinch grafting was done by a standard method.[1] Eight pinches were taken from the right thigh and placed over the ulcer [Figure 2]. All the grafts survived and ulcer healed after 6 weeks [Figure 3].

## DISCUSSION

Principles of management of chronic venous leg ulcers include reduction of oedema, alleviation of pain, healing of ulcers, improvement of lipodermatosclerosis and prevention of recurrence.[2] Bed rest and leg elevation reduces the oedema and speeds up healing in venous stasis ulcer. Multiple positivity to patch test is well known in chronic leg ulcers.[3] Compression remains the cornerstone of therapy for patients with more advanced venous ulcers. In-elastic rigid bandages are ideal in acute phase, as they mostly act as a support system. The bandage exerts minimum pressure at rest and pressure increases with muscle contraction. The prototype of this bandage is the traditional Unna boot, moist zinc impregnated paste bandage using zinc oxide and gelatin.[4] Unna boot also reduces pain.[5] We used a modified Unna boot using 40% zinc oxide and 60% glycerin paste, since gelatin gets solidified immediately and is difficult to transport it from pharmacy to bed side. Patients with a larger wound area, greater duration of wound, history of venous ligature, venous stripping and history of hip or knee replacement are the best candidates for compression therapy with Unna boot.[2]

Pinch grafting is the first option, as it is a simple, effective and can be performed in the office.[6] Skin grafting should be considered in large and refractory leg ulcers.

We report this case as we combined the traditional Unna boot which is not commonly practiced nowadays and pinch grafting which is a simple effective surgery to rapidly rehabilitate the patient.
